# A Water Environment-Based Simulated Method for Ultrasonic Testing of Slag Inclusion Weld Defects Based on Improved VMD

**DOI:** 10.3390/s24134199

**Published:** 2024-06-28

**Authors:** Jing Zhang, Guocai Zhang, Zijie Chen, Hailin Zou, Shuai Xue, Jianjie Deng, Jianqing Li

**Affiliations:** 1 School of Computer Science and Engineering, Faculty of Innovation Engineering, Macau University of Science and Technology, Macau 999078, China; 2009853gii30017@student.must.edu.mo (J.Z.);; 2 School of Applied Science and Civil Engineering, Beijing Institute of Technology, Zhuhai 519006, China; 3 School of Mechanical and Automotive Engineering, South China University of Technology, Guangzhou 510640, China

**Keywords:** slag inclusion, ultrasonic testing, weld defects, variational mode decomposition, particle swarm optimization

## Abstract

The identification of slag inclusion defects in welds is of the utmost importance in guaranteeing the integrity, safety, and prolonged service life of welded structures. Most research focuses on different kinds of weld defects, but branch research on categories of slag inclusion material is limited and critical for safeguarding the quality of engineering and the well-being of personnel. To address this issue, we design a simulated method using ultrasonic testing to identify the inclusion of material categories in austenitic stainless steel. It is based on a simulated experiment in a water environment, and six categories of cubic specimens, including four metallic and two non-metallic materials, are selected to simulate the slag materials of the inclusion defects. Variational mode decomposition optimized by particle swarm optimization is employed for ultrasonic signals denoising. Moreover, the phase spectrum of the denoised signal is utilized to extract the phase characteristic of the echo signal from the water–slag specimen interface. The experimental results show that our method has the characteristics of appropriate decomposition and good denoising performance. Compared with famous signal denoising algorithms, the proposed method extracted the lowest number of intrinsic mode functions from the echo signal with the highest signal-to-noise ratio and lowest normalized cross-correlation among all of the comparative algorithms in signal denoising of weld slag inclusion defects. Finally, the phase spectrum can ascertain whether the slag inclusion is a thicker or thinner medium compared with the weld base material based on the half-wave loss existing or not in the echo signal phase.

## 1. Introduction

Austenitic stainless steel is widely preferred by industries and used in media storage, transportation, processing and production, equipment manufacturing, and other industries due to its mechanical properties (such as high tensile strength, good yield strength, and high-temperature strength), outstanding corrosion resistance, and good weldability [1]. Currently, welding is fusing metal parts to form a strong bond that can withstand harsh conditions. The quality of the weld is critical to safety and performance, as defects can lead to failures, accidents, and financial losses [2]. Weld inspection is an important task in non-destructive testing (NDT), which is necessary to detect weld defects. During the welding process, defects can occur on a weldment, such as porosity, gas pores, longitudinal cracks, lack of penetration, and slag inclusion [3]. Slag inclusion in these defects means volume defects not as visible as cracks, incomplete penetration, or incomplete fusion defects, which are called surface defects.

Defect testing of weldments has attracted much attention in recent years. Commonly used weld defect detection methods include eddy current [4], magnetic particle inspection [5], X-ray [6], spectroscopy emission [7], and ultrasonic testing (UT) [8]. Ghubade and Kumar [9], in their review work on casting defects and methodologies for quality improvement, provided insight into casting defects and casting quality. Singh et al. [10] proposed an active pulse infrared thermal imaging technology detection method which uses a FlukeTi 32 thermal imaging camera to record the metal casting heating and temperature decay processes. Using the prior knowledge that defect-containing regions have higher temperatures than defect-free regions, defect regions with high thermal concentrations and the size and depth of defects are characterized. Li et al. [11] proposed a frequency band-selecting pulsed eddy current testing (FSPECT) method. Compared with energy equivalent square wave PEC technology, FSPECT responds better and faster to deep defects. The authors of [12] designed an active thermal imaging system with induction heating. By recording the surface temperature with an infrared thermal imaging camera, the entire infrared image sequence is evaluated using a Fourier transform, and the phase image is processed to detect surface cracks. At the same time, the signal distribution around the cracks at different depths is analyzed and calculated to determine the crack depth. Wang et al. [13] designed a digital ray detection system based on charge-coupled devices. The system can receive X-rays with a wider energy range and obtain higher imaging quality. Tkocz et al. [14] designed a low-cost four-channel phased array electromagnetic ultrasonic transducer system. The generated focused ultrasonic beam completed the defect detection experiment on a rough cast steel plate with a thickness of 32 cm. A-scan data showed that the system could detect artificial defects up to a depth of 16 cm.

The research mentioned above primarily focused on the size and depth of internal defects in materials. However, the literature on the different categories of slag inclusion defects is limited. Slag inclusion defects are typically classified as metal or non-metal inclusions. Slag defects arise from multiple factors, including suboptimal welding practices, inadequate preheating, and imprecise control over welding parameters. It is difficult to determine the accuracy of the test results for slag inclusion types since the process is irreversible and destructive to the specimen. The process of producing austenitic stainless steel is highly intricate and demands a controlled production environment. This poses a challenge when it comes to obtaining slag inclusion defect samples with known slag material. Numerical simulated methods based on finite element analysis have attracted attention from many researchers [15,16]. This simulation of the ultrasonic testing system could easily design or optimize an ultrasonic testing system by regulating the parameters of the system, which offers a theoretical foundation for detection and reduces detection blindness [17]. However, the effectiveness of this simulation approach often tends to be overly ideal. Therefore, we designed a water environment-based experiment for slag inclusion defect detection to address these issues.

Radiographic testing (RT) can be implemented utilizing either X-ray radiation or a gamma-emitting radioactive source [18]. Given the pronounced dependence of the X-ray absorption coefficient on the material density, radiographic imaging is particularly well suited for identifying volume defects characterized by an altered mass content, such as slag inclusions or porosity [19]. RT offers a more direct and precise determination of the material type of slag inclusions within welds. However, it is limited by the harmful radiation it generates, the complexity of the detection process, and the extended duration of the testing, which preclude its suitability for in situ, real-time detection in industrial environments, such as the scenario of steel structure weld inspection for a building. Compared with the RT method, UT can overcome these disadvantages and is more widely applied in weld inspections due to its safety, portability, in situ and real-time feedback, and low cost. Moreover, UT can test the depth information of a defect. UT is a commonly used method for detecting flaws in solids. It provides precise information about the position and size of defects. Therefore, we chose UT in this paper to detect the inclusion defects. However, ultrasonic signals can be easily scattered by complex factors such as the environment and the object being measured. This often leads to noise in the received echo signal, which can affect the quality and reliability of the measurement. Therefore, denoising is an essential step for removing noise and enhancing the signal. Classical signal denoising algorithms include wavelet transform (WT) [20], empirical mode decomposition (EMD) [21], and its variants such as ensemble EMD (EEMD) [22] and complementary EEMD with adaptive noise (CEEMDAN) [23]. EMD series methods may produce mode mixing or cause incomplete signal decomposition and denoising with residual noise in the denoised signal. Variational mode decomposition (VMD) is a new algorithm for decomposing signals into several intrinsic mode functions (IMFs) with different center frequencies to pick out the effective signal [24]. It overcomes the problems of endpoint effects and modal aliasing in EMD [25]. Compared with other methods, VMD has been proven to yield a better frequency resolution and noise immunity [26]. However, it requires manually setting the hyperparameters. To address this issue, researchers have investigated methods for setting parameters automatically. For instance, Hua et al. applied the grasshopper optimization algorithm (GOA) to further fine-tune the parameters in VMD for Lidar signal decomposition [27]. Similarly, Qi et al. harnessed the grey wolf optimization (GWO) method to determine the most effective VMD parameters, alongside parallel EMD for Lidar signal noise reduction [28]. Additionally, Long et al. made use of particle swarm optimization (PSO) to identify the ideal VMD parameters for filtering out disturbances from ultra-high-frequency partial discharge signals [29].

In the ultrasonic detection of slag inclusion defects in austenitic steel welds, the signal will exhibit a superior signal-to-noise ratio after effective denoising. A Fast Fourier transform (FFT) is performed on the denoised signal to obtain the phase spectrum. In accordance with the principles of wave propagation theory, it is established that mechanical and electromagnetic waves, when encountering an interface with a transition to a denser medium, experience a π phase shift upon reflection. This is in contrast to the situation where they interact with a less dense medium, where no such phase change occurs. This intrinsic relationship between the echo phase and the characteristics of the interface material is invariant across diverse experimental settings and environmental conditions. Consequently, we introduce a novel experimental methodology for the ultrasonic detection of slag inclusion defects in welds, which is based on the simulation of aquatic environments.

In a nutshell, we designed a series of experiments utilizing selected cubic specimens made of various materials immersed in a water-based environment to simulate different material types in slag inclusion defects found in weldments. Specifically, the six selected cubes were composed of different materials, including metallic materials such as iron, copper, aluminum, and magnets, as well as non-metallic materials like wood and resin. The primary objective was to analyze the phase characteristic information of ultrasonic signals and investigate the relationship between phase and slag inclusion materials by utilizing ultrasonic testing in conjunction with an improved VMD denoising method, thereby achieving the classification and detection of slag inclusion defects.

The rest of this paper is organized as follows. The methodology employed in this study is outlined in Section 2; the experiments are shown in Section 3; and the results analysis is discussed in Section 4. Finally, this work is summarized in the Section 5.

## 2. Theory and Methods

### 2.1. VMD

#### 2.1.1. VMD Theory

VMD is a non-recursive variational mode decomposition algorithm showing excellent capacity for processing nonlinear and non-stationary signals which was proposed by Dragomiretskiy [24]. It ensures sparsity by limiting the central frequency and bandwidth of each IMF. VMD can avoid issues related to mode mixing and boundary effects, which always exists in EMD, WT, etc. The constrained model is defined as follows:

(1) $$\left\{\begin{array}{c} \min_{\left(u_{k},\omega_{k}\right)}\left\{\sum_{k=1}^{K}{∥\partial_{t}\left[\left(\sigma \left(t\right)+\frac{j}{\pi t}\right)⊗u_{k}\left(t\right)\right]e^{-j\omega_{k}t}∥}_{2}^{2}\right\}\\ \;s.t.\;\sum_{k=1}^{K}u_{k}\left(t\right)=f\left(t\right) \end{array}\right.$$

where $u_{k}\left(t\right)$ represents the kth IMF, $\omega_{k}$ represents the central frequency of $u_{k}\left(t\right)$, k=1,2,⋯,K, σ(t) is the unit pulse function, ⊗ represents a convolutional computation, and $\partial_{i}$ represents a partial derivative operation.

Initially, the Hilbert transform was applied to each IMF to obtain a one-sided spectrum:

(2) $$\left(\sigma \left(t\right)+\frac{j}{\pi t}\right)⊗u_{k}\left(t\right).$$

Then, we estimated the central frequency ($\omega_{k}$) of each IMF and modulated each IMF to the baseband:

(3) $$\left[\left(\sigma \left(t\right)+\frac{j}{\pi t}\right)⊗u_{k}\left(t\right)\right]e^{-j\omega_{k}t}.$$

Lastly, the bandwidth of each IMF was estimated through the $H_{1}$ Gaussian smoothness by calculating the $L^{2}-norm$ of the demodulated signal gradient.

#### 2.1.2. Solution of Variational Equations

The equation initially presented as Equation (1) was converted into a form without constraints by employing the Lagrange multiplier, denoted by λ. Furthermore, to maintain the fidelity of signal reconstruction in the presence of additive Gaussian noise, a quadratic penalty factor, represented by α, was utilized. The resulting unconstrained formulation is expressed as follows:

(4) $$\begin{array}{cc} L\left(\left\{u_{k}\right\},\left\{\omega_{k}\right\},\lambda \right) & =\alpha \sum_{k}{∥\partial_{t}\left[\left(\sigma \left(t\right)+\frac{j}{\pi t}\right)⊗u_{k}\left(t\right)\right]e^{-j\omega_{k}t}∥}^{2}+{∥f\left(t\right)-\sum_{k}u_{k}\left(t\right)∥}_{2}^{2}+<\lambda \left(t\right),f\left(t\right)-\sum_{k}u_{k}\left(t\right)> \end{array}$$

Next, by solving Equation (4), $\hat{f}\left(\omega \right)$ and ${\hat{\omega }}_{k}$ are updated as follows:

(5) $${\hat{u}}_{k}^{n+1}\left(\omega \right)=\frac{\hat{f}\left(\omega \right)-\sum_{i\neq k}{\hat{u}}_{i}\left(\omega \right)+\frac{\hat{\lambda }\left(\omega \right)}{2}}{1+2\alpha {\left(\omega -\omega_{k}\right)}^{2}}$$

(6) $$\omega_{k}^{n+1}=\frac{\int_{0}^{\infty }\omega {\left|{\hat{u}}_{k}\left(\omega \right)\right|}^{2}\;d\omega }{\int_{0}^{\infty }{\left|{\hat{u}}_{k}\left(\omega \right)\right|}^{2}\;d\omega }$$

where ${\hat{u}}_{i}\left(\omega \right)$, $\hat{f}\left(\omega \right)$, $\hat{\lambda }\left(\omega \right)$ are the Fourier transform of $u_{k}\left(t\right)$, f(t), $\lambda \left(t\right)$ respectively.

The VMD algorithm’s process has four main steps:

- Step 1. Initialize $u_{k}^{1}$, $\omega_{k}^{1}$, $\lambda_{k}^{1}$, and *n* such that all their values are zero, and set a suitable number of decomposition modes *K*.
- Step 2. Iteratively update $u_{k}$ and $\omega_{k}$ using Equations (5) and (6), and stop when the preset value *K* is reached.
- Step 3. Update λ using the following equation:
  (7) $${\hat{\lambda }}^{n+1}\left(\omega \right)\leftarrow {\hat{\lambda }}^{n}\left(\omega \right)+\tau \left(\hat{f}\left(\omega \right)-\sum_{k}{\hat{u}}_{k}^{n+1}\left(\omega \right)\right)$$
  where τ represents the noise tolerance for a given discrimination accuracy $0<\phi <1\times 10^{-7}$. If this satisfies the following condition, then stop the iteration. Otherwise, return to Step 2:
  (8) $$\begin{array}{c} \sum_{k=1}^{K}\frac{∥{\hat{u}}_{k}^{n+1}-{\hat{u}}_{k}^{n}{∥}_{2}^{2}}{∥{\hat{u}}_{k}^{n}{∥}_{2}^{2}}=\phi \end{array}$$
  where ${\hat{u}}_{k}$ is the updated expression of $u_{k}$.
- Step 4. Arrange the obtained *K* IMF components from low to high frequencies, calculate the correlation coefficient between each component and the original signal, eliminate the IMF components with smaller correlation coefficients, and reconstruct the remaining components.

### 2.2. Improved VMD through Particle Swarm Optimization

Efficient and precise parameter selection for VMD hinges on the choice of (*K*, α). This study employs particle swarm optimization to ascertain the optimal (*K*, α) values for VMD, thereby eliminating subjective judgment and automating the search for the best parameters. The fundamental concept behind employing the PSO algorithm to discover the best solution lies in the collaborative effort and exchange of information among the various members of the population [30]. The algorithm can be described as follows.

In an *N*-dimensional space, a swarm consists of *m* particles, where $x_{i}=\left(x_{i1},x_{i2},\cdots ,x_{in}\right)$ represents the position of the ith particle in the *N*-dimensional space, $v_{i}=\left(v_{i1},v_{i2},\cdots ,v_{in}\right)$ represents the motion of a particle, $p_{i}=\left(p_{i1},p_{i2},\cdots ,p_{in}\right)$ represents the local optimal solution that the particle has experienced, and $p_{g}=\left(p_{g1},p_{g2},\cdots ,p_{gn}\right)$ is the best position among the entire swarm as the global optimum.

In PSO, the update of velocity and position of a particle can be calculated as follows:

(9) $$v_{id}\left(t+1\right)=\omega v_{id}\left(t\right)+c_{1}r_{1}\left[p_{id}-x_{id}\left(t\right)\right]+c_{2}r_{2}\left[p_{gd}-p_{gd}\left(t\right)\right]$$

(10) $$x_{id}\left(t+1\right)=v_{id}\left(t\right)+v_{id}\left(t+1\right)$$

where ω is the inertia weight, $c_{1},c_{2}$ are the learning factors, and $r_{1},r_{2}$ are random numbers distributed in the range [0, 1].

Specifically, the optimal parameter (*K*, α) is selected in parallel by introducing a two-dimensional particle swarm. According to Equations (9) and (10), there will be the following equations:

(11) $$\begin{array}{c} \left[\begin{array}{c} v_{k}\left(t+1\right)\\ v_{\alpha }\left(t+1\right) \end{array}\right]=\omega \left[\begin{array}{c} v_{k}\left(t\right)\\ v_{\alpha }\left(t\right) \end{array}\right]v_{id}\left(t\right)+c_{1}r_{1}\left(\left[\begin{array}{c} p_{k}\\ p_{a} \end{array}\right]-\left[\begin{array}{c} x_{k}\left(t\right)\\ x_{\alpha }\left(t\right) \end{array}\right]\right)+c_{2}r_{2}\left(\left[\begin{array}{c} g_{k}\\ g_{\alpha } \end{array}\right]-\left[\begin{array}{c} x_{k}\left(t\right)\\ x_{\alpha }\left(t\right) \end{array}\right]\right) \end{array}$$

(12) $$\left[\begin{array}{c} x_{K}\left(t+1\right)\\ x_{\alpha }\left(t+1\right) \end{array}\right]=\left[\begin{array}{c} x_{K}\left(t\right)\\ x_{\alpha }\left(t\right) \end{array}\right]+\left[\begin{array}{c} v_{K}\left(t+1\right)\\ v_{\alpha }\left(t+1\right) \end{array}\right].$$

The intricacy of components indicates their regularity, with reduced complexity signifying a higher degree of regularity. The sample entropy serves as a statistical instrument for assessing the complexity and unpredictability of time series data [31]. Essentially, the sample entropy measures the probability of recurrence of comparable data patterns in a time series. Consequently, a greater sample entropy value suggests a more intricate, less predictable, and more irregular time series.

The equation for the sample entropy is given below:

(13) $$SampEn\left(m,r,N\right)=-ln\left[\frac{B^{m+1}\left(r\right)}{B^{m}\left(r\right)}\right]$$

where *r* represents the signal tolerance, *m* is the embedding dimension, *N* is the signal length, and $B^{m}\left(r\right)$ is the mean pattern count.

The fitness function described in this paper, represented by Equation (14), is calculated as the average of the squared error (SE) for all intrinsic mode functions (IMFs) obtained from a single variational mode decomposition (VMD). A fitness value of less than 0.1 indicates that the optimal solution has been achieved:

(14) $$fitness\left(K,\left\{u_{k}\right\}\right)=\frac{1}{K}\sum_{k=1}^{K}SampEn\left(u_{i}\right)$$

where *K* is the total number of IMFs decomposed via VMD and $\left\{u_{k}\right\}=\left\{u_{1},\cdots ,u_{K}\right\}$ are IMFs.

PSO can be optimized by minimizing the fitness function with K and alpha as the optimization-seeking parameters. The following steps should be taken:

- Step 1. Firstly, input the original series (ultrasonic signal). Then, set the range of the VMD parameter pairs (*K*, α), where *K* takes integer values in the range of [2, 12] and α’s range is within [100, 30,000]. After that, initialize the positions and velocities of the swarm’s particles. Finally, calculate the local and global optimal solutions using Equation (14).
- Step 2. Apply Equations (11) and (12) to update the particle positions. Refresh the local and global solutions accordingly.
- Step 3. Output the global optimal solution of the IMF’s number *K* and the penalty factor α when the fitness value is less than 0.1 or the maximum number of iterations is reached.
- Step 4. Using the optimized results, apply the VMD method to break down the vibration signal into different IMFs. Filter out the IMFs that fall outside the frequency range of the ultrasonic processing equipment, and combine the remaining effective IMFs to obtain a precise ultrasonic vibration signal.

### 2.3. Ultrasonic Wave Propagation Theory

Classical wave propagation theory posits that a wave transitioning from a less dense to a denser medium will undergo reflection at the point of intersection between the two media. The cause of this reflection is the abrupt phase shift of the reflected wave at the midpoint of its cycle, a concept referred to as half-wave loss [32]. Half-wave loss is a phenomenon that occurs when a mechanical wave reflects at the interface between two media with different properties. This typically happens when the wave transitions from a less dense medium to a denser one, such as from air to a solid. In the case of mechanical waves like ultrasound, this occurs when the wave transitions from a medium with a lower refractive index to one with a higher refractive index. The reflected wave undergoes a phase shift of pi, which is equivalent to a half-wavelength change in the spatial period. This phase shift leads to a so-called phase inversion, where the wave phase becomes negative after reflection if it is positive before reflection at the interface between the media.

Half-wave loss in the phase of the ultrasonic echo will occur at the interface when propagation is constituted in a wave-dense medium compared to steel, such as the tungsten inclusion defect. However, the phase of ultrasonic echo will change continuously when ultrasonic waves propagate through the steel matrix and encounter porosity or non-metallic inclusions, and a reflection will occur at the interface between the steel and non-metallic inclusion without the half-wave loss. Depending on the phase of the ultrasonic echo, we can determine whether the inclusions of the slag are a thin medium or thick medium relative to the steel matrix.

Welding slag inclusions are embedded within the austenitic stainless steel matrix, serving as a model for an environment with a foreign object included. Foreign objects can be categorized as either wave-thin or wave-dense media relative to the matrix material [33]. According to wave propagation theory, ultrasonic waves propagate through the environment and reflect off foreign objects. The presence or absence of a half-wave loss in the echo signal’s phase can indicate whether the foreign material is a wave-thin or wave-dense medium. In our experimental set-up, water and diverse material samples were employed to simulate the environment and slag inclusions, respectively.

## 3. Experiment

### 3.1. Experimental Set-Up

**Figure 1:**
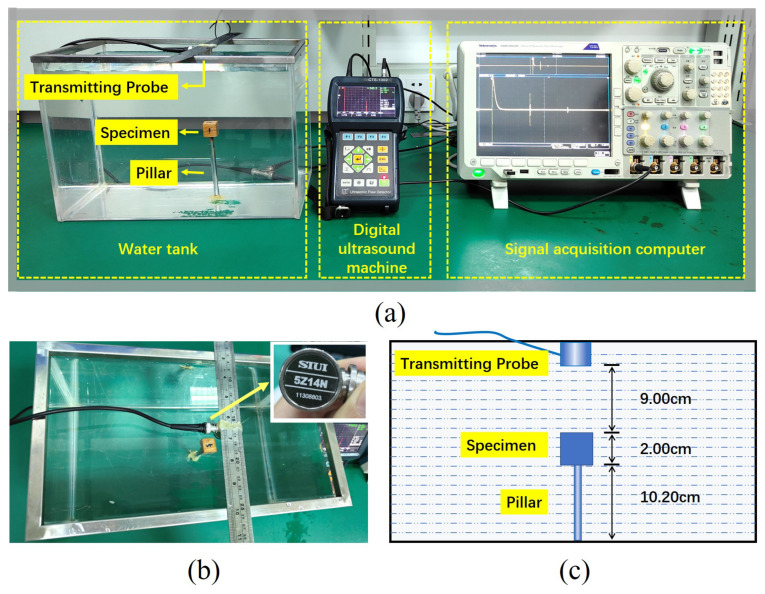
(**a**) Experimental system of ultrasonic testing based on water environment simulation. (**b**) Top view of the water tank and ultrasonic probe. (**c**) Schematic diagram of vertical distances between objects placed inside the water tank.

**Figure 2:**
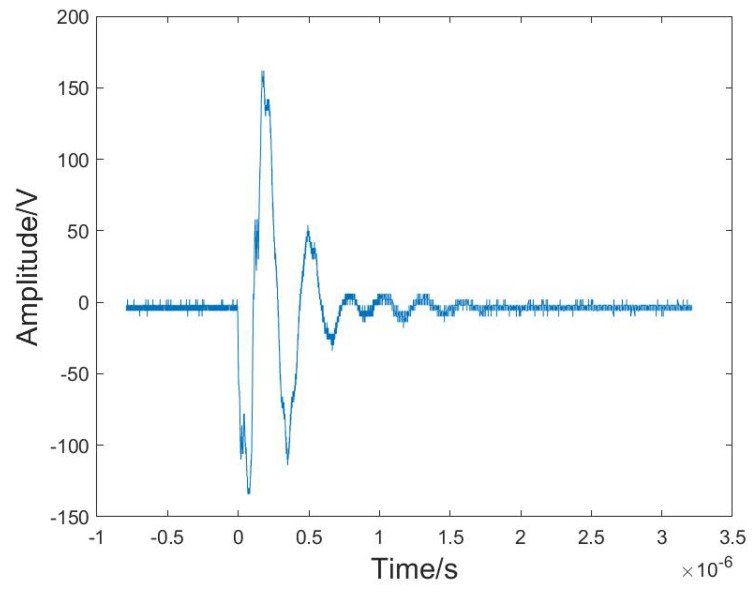
The loaded excitation waveform of the probe with the SIUI CTS–1002 operating at a working voltage of 100 V.

As it is not easy to observe the material of slag inclusion in weld defects, this experiment utilized a simulated experiment with a water environment to simulate defects inside the weldment, as shown in Figure 1a. The ultrasound experimental system consisted of an SIUI CTS-1002^®^ digital ultrasound machine (Shantou, China), a 5Z14N transmitting probe (SIUI longitudinal wave straight probe, 5 MHz, 14 mm, shown in Figure 1b), and a signal acquisition computer (Tektronix MDO4024C oscilloscope, Beaverton, OR, USA). The ultrasonic probe was responsible for transmitting and receiving the ultrasonic signals, producing pulsed ultrasonic signals at a nominal center frequency of 5 MHz. Theoretically, its operating frequency would be equal to the nominal frequency. However, a downshift in frequency could be potentially caused by the manufacturing technology of the piezoelectric crystal probe and aging of the detector with a served time duration. Figure 2 illustrates the loaded excitation waveform of the probe when the SIUI CTS-1002 was operating at a voltage of 100 V. The oscilloscope operated at a sampling frequency of 2.5 GHz, which was employed to visualize the temporal disparity of ultrasonic waves as they traversed various positions within austenitic stainless steel. It filled the tank with water, fixed a cylindrical pillar on the bottom, and placed the cubic sample on top of the pillar in this experiment. We maintained a certain distance between the sample and the bottom of the water tank to distinguish the echo signals reflected from the sample surface and the bottom of the tank. Figure 1c shows that the vertical distances were 9.00 cm and 10.20 cm between the upper surface of the slag specimen and the transmitting probe surface and the bottom surface of the specimen and the bottom of the water tank, respectively. The specimen was a cubic sample with a side length of 2.00 cm. The ultrasonic probe was placed on the top of the water tank to transmit ultrasound vertically and directly immerse it in the water, which eliminated the need for a coupling agent and resulted in reduced attenuation of the ultrasound wave. The reflected ultrasonic echo signals of the 6 cubic specimens were collected by our ultrasound system.

**Figure 3:**
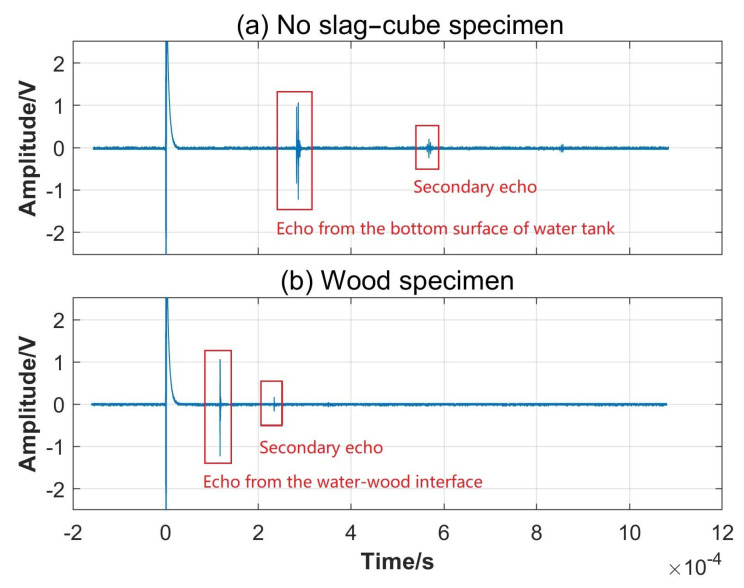
Original echo signals measured by the oscilloscope: (**a**) no slag cube specimen and (**b**) wood cube specimen placed in the water environment.

The original echo signals were collected by the oscilloscope shown in Figure 3, where (a) is the reference original echo signal obtained when no slag cube block was placed in the water environment and (b) is the original echo signal obtained when a wood specimen was immersed in the water tank. The signal encircled by a red box on the left in Figure 3a is the echo from the bottom surface of the water tank, and the following weaker one encircled by the right red box is the secondary echo. It can be seen that the first echo’s occurrence time was approximately $2.8\times 10^{-4}$ s, given an ultrasonic wave propagation velocity of approximately 1500 m/s. This corresponded to a traveled distance of approximately 42 cm, which was consistent with twice the distance between the ultrasonic probe and the bottom surface of the water tank (21.20 cm), as shown in Figure 1c. Upon placement of a slag specimen in the water, the ultrasonic waves underwent multiple traversals between the ultrasonic detector and specimen. As depicted in Figure 3b, the red rectangles highlight the initial and secondary echoes reflected from the water–wood cubic block interface, occurring at approximately $1.2\times 10^{-4}$ s and $2.4\times 10^{-4}$ s, respectively. These echo arrival times corresponded to the distances traversed by the ultrasonic waves, in accordance with the experimental distance set-up of 9.00 cm between the transmitting probe and slag inclusion specimen.

**Figure 4:**
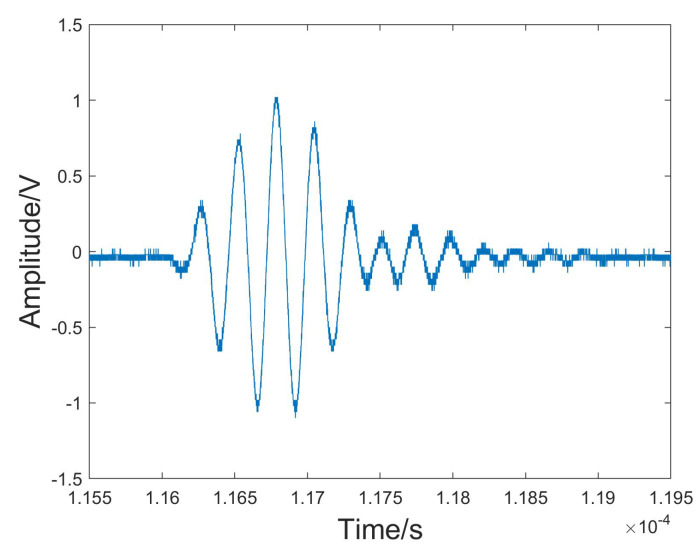
The original echo signal reflected from the water–wood interface.

The original echo signal reflected from the sample surface was intercepted and used for analysis in this experiment. We chose the experiment of the wood specimen immersed in water as an example to illustrate the experimental procedures and subsequent analysis of the results. The chosen white birch wood specimen had a density of approximately 0.599 g/cm^3^ in a dry state and approximately 0.676 g/cm^3^ after being submerged in water for half an hour. An original ultrasound echo signal from the sample of wood is shown in Figure 4, VMD was performed on the ultrasonic echo signal. As can be seen in the figure, there was significant noise in the signal, especially at the beginning and end of the signal.

### 3.2. Evaluating Indicator

Signal denoising is the extraction of effective information from the original signal. VMD is an adaptive decomposition method. After decomposition by these algorithms, a series of IMFs with a certain center frequency can be obtained. One or more layers of the decomposed IMF contain noise for noisy signals. Moreover, it is particularly important to judge whether the IMF is noise or a useful signal. Usually, in the signal processing process, some researchers will directly discard IMFs with smaller orders. At the same time, some useful signals contained in IMFs with smaller orders will also be discarded. However, the signal-to-noise ratio (SNR) is reduced, and the denoising effect is poor. This paper first obtains the normalized cross-correlation (NCC) function of each modal function IMF and the original signal and then uses the obtained NCC to determine the useful signal mode and noise mode.

In this case, we needed to select effective IMFs for reconstruction to obtain denoising signals. The SNR and RMSE were used as evaluation indices of the denoising effect. The SNR shows an energy relationship between the signal and noise. The higher the SNR, the more useful the information, and the less noise there is in the signal. Therefore, the SNR can be a quite intuitive method for evaluating the effect of denoised signals by analyzing whether the SNR improves. The definition of the SNR is as follows:

(15) $$SNR=10\cdot \log_{10}\left(\frac{\sum_{i=1}^{N}{∥x\left(i\right)∥}^{2}}{\sum_{i=1}^{N}∥\hat{x}{\left(i\right)-x\left(i\right)∥}^{2}}\right)$$

where *x* is the original signal, $\hat{x}$ is the denoised signal, and ∥∗∥ indicates the norm.

The root mean square error shows the difference between the denoised signal and original signal in numerical terms, and the smaller the RMSE, the better the noise reduction effect. The RMSE is defined as follows:

(16) $$RMSE=\sqrt{\frac{\sum_{i=1}^{N}{∥\hat{x}\left(i\right)-x\left(i\right)∥}^{2}}{\mathrm{length}(x)}}$$

where length(∗) represents the length of the signal.

In order to effectively identify and separate the noise component in the IMFs decomposed by VMD, a normal correlation coefficient was introduced based on time–frequency analysis of the IMF component to further determine the degree of correlation between the IMF and the original signal. The larger the NCC, the better the correlation between the corresponding IMFs and the original signal. The NCC is defined by

(17) $$NCC=\frac{\sum_{i=1}^{N}\left(u_{k}\left(i\right)\cdot \hat{x}\left(i\right)\right)}{\sqrt{\left(\sum_{i=1}^{N}u_{k}^{2}\left(i\right)\right)\times \left(\sum_{i=1}^{N}{\hat{x}}^{2}\left(i\right)\right)}}$$

where $u_{k}$ and $\hat{x}$ are the kth IMF and denoised signal, respectively.

## 4. Results

### 4.1. PSO-VMD Denoising

This section presents the results of the echo signal decomposition received from the water–slag specimen interface through VMD optimized by PSO. To validate the superiority of our proposed vibration signal processing method, we compared it with other methods. The comparative methods included WOA [34], GA [35] and ALO [36] algorithms used to optimize the two core parameters (*K*, α) of VMD. The defect signal processing of ultrasonic testing data from the water–wood interface served to illustrate the application of the IMF’s section approach in the proposed PSO-VMD method. We applied these methods to process the original ultrasonic echo signals individually. The fundamental concept underlying these methods is to decompose the original defect signal and eliminate non-ultrasonic components. By employing the PSO technique for parameter optimization adaptively, we deployed VMD on the ultrasonic defect testing data.

To compare the accuracy and efficiency of the different signal processing methods, Table 1 lists the main parameters *K* and α for the improved VMD algorithms, SNR, RMSE, and processing time. The SNR and RMSE indicators were employed to assess the approaches’ accuracy, while the processing time refers to quantitatively analyzing the efficiency of denoising. It can be seen that all of the improved VMD methods could decompose the original signal by setting the parameters *K* and α automatically. The proposed PSO-VMD method provided the least IMFs (seven) with the highest SNR and the lowest RMSE. The SNR reflects the ratio of the overall signal to the noise component, where the higher the SNR, the better the noise reduction effect. The RMSE quantitatively represents the deviation between the denoised signal and the original signal, where a lower RMSE indicates more effective noise reduction. PSO-VMD could effectively denoise and extract features from ultrasonic testing signals to highlight the slag inclusion defect features. Compared with PSO-VMD, the WOA-VMD, GA-VMD and ALO-VMD methods demonstrated varying extents of over-decomposition phenomena, which decomposed the signal into 10, 8, and 10 IMFs, respectively.

Additionally, a comparison experiment on the denoising efficiency of the improved VMD algorithms was conducted on a PC equipped with an Intel i7-3700K CPU (Santa Clara, CA, USA), Nvidia Geforce RTX 3090 GPU, and 128 GB of memory (Santa Clara, CA, USA). Each algorithm was configured with the maximum number of iterations set to 10, and each was executed 10 times on the echo signals of wood specimen. The average processing times for each algorithm are presented in Table 1. The experimental results indicate that both the WOA and ALO optimization algorithms exhibited superior efficiency, with processing times of 79.7 s and 78.9 s, respectively, in comparison with the GA method’s 1155.9 s, demonstrating the lowest efficiency. Our proposed PSO algorithm held a middle ground at 536.7 s in terms of efficiency. Consequently, there exists a considerable scope for enhancement in the noise reduction efficacy of PSO-VMD when compared with the WOA-VMD and ALO-VMD algorithms. It is anticipated that advancements in computational power will mitigate this discrepancy, rendering it a non-obstacle in the foreseeable future.

**Figure 5:**
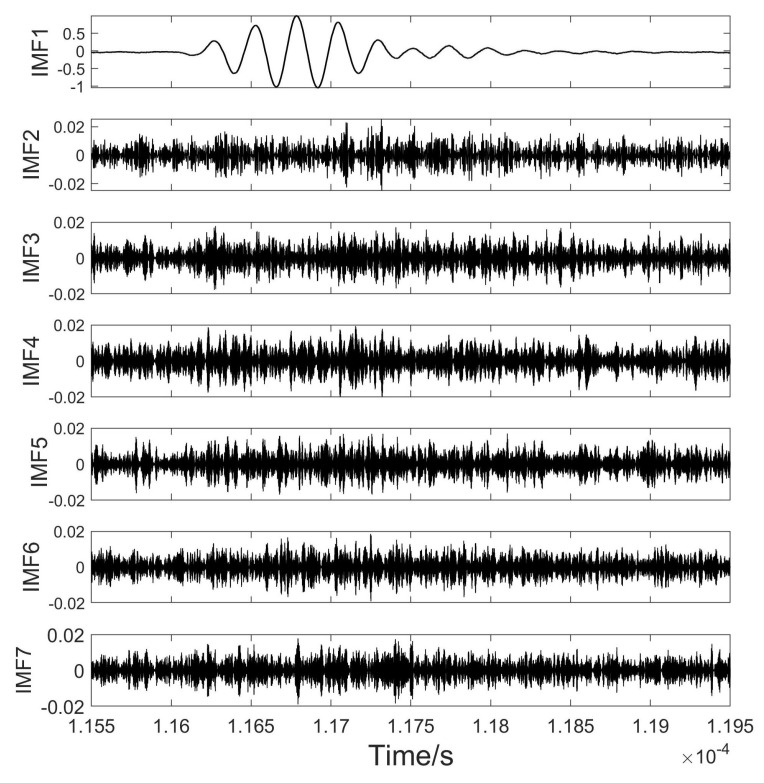
PSO–VMD decomposition results in time domain.

**Figure 6:**
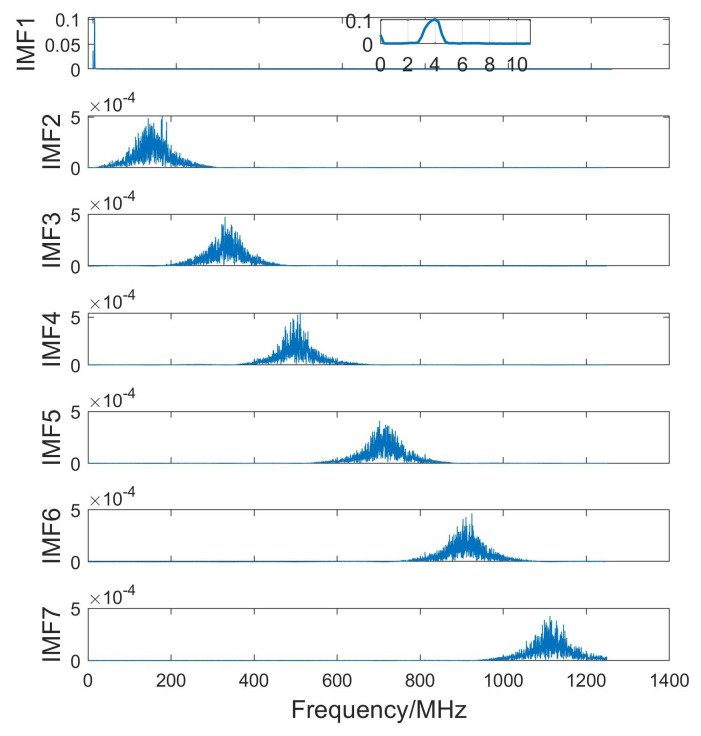
The decomposed IMF spectrum under PSO–VMD in the frequency domain. The inset in IMF1 is a local enlargement of the IMF component.

The components were sorted by frequency from low to high in Figure 5 for IMFs in the time domain and in Figure 6 for IMFs in the frequency domain. The low-frequency component IMF1 demonstrated strong periodic fluctuations, and the high-frequency component appeared to be random. IMF1 symbolized effective signals from slag inclusion defects, while other IMF components corresponded to interference or noise. The echo signals from the slag specimens could be effectively distinguished from the interference components.

**Figure 7:**
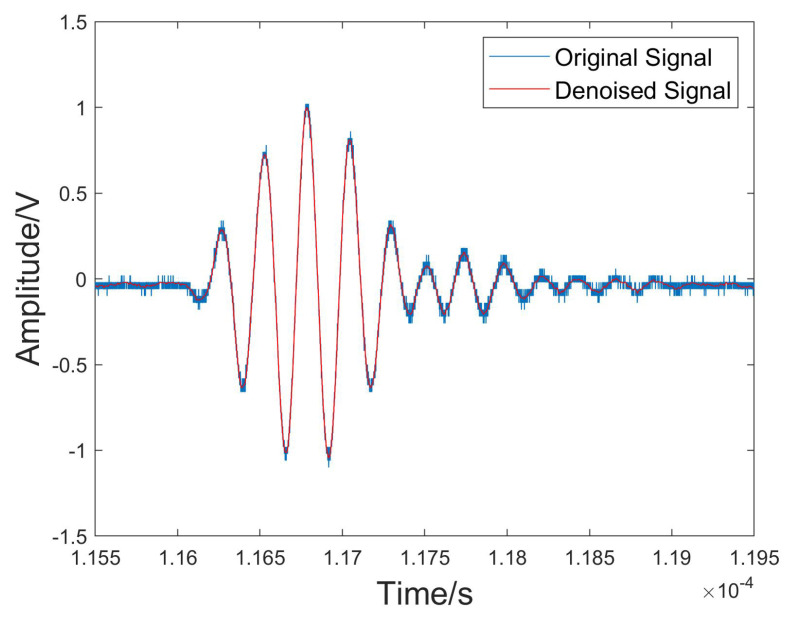
Denoised signal and original signal from the water–wood interface.

Table 2 shows the normalized cross-correlation between the IMF components of the denoised signal and the original signal. The NCC of IMF1 was 0.9975, which was significantly higher than those of the other modes. It can be seen from the spectrogram presented in Figure 6 that the center frequencies among the IMFs were distinct and separate without any overlap, reflecting the efficacy of PSO in the context of VMD parameter tuning. Figure 7 shows the comparison between the denoised signal and the original signal, which exhibited characteristics similar to the original data but with less noise and a higher signal-to-noise ratio.

### 4.2. Phase of the Echo Signal

In this experiment, water was the original medium for ultrasonic wave propagation, and the surfaces, those of cubic blocks of iron, aluminum, copper, wood, resin, and other materials placed in the water environment to simulate slag inclusion defects, were the interfaces for sound wave reflection. Among the six materials selected, metallic blocks such as iron, aluminum, copper, and magnets are thick media compared with water, while non-metallic wood and resin are thin media. By analyzing the phase spectrum of the echo signal, we could determine whether the simulated inclusion was a thin medium or a thick one based on the half-wave loss theory.

The phase spectrum describes the phase information of the frequency components within a signal. In practical applications, the phase spectrum helps with understanding the phase relationships among different frequency components of a signal, which is quite important for signal analysis, processing, and interpretation. According to the theory of half-wave loss, in the propagation of sound waves, the phase spectrum serves as a critical means for analyzing reflected signals. We could determine whether the material at the reflective interface was a denser medium by assessing whether the reflected signal had undergone a π phase shift.

**Figure 8:**
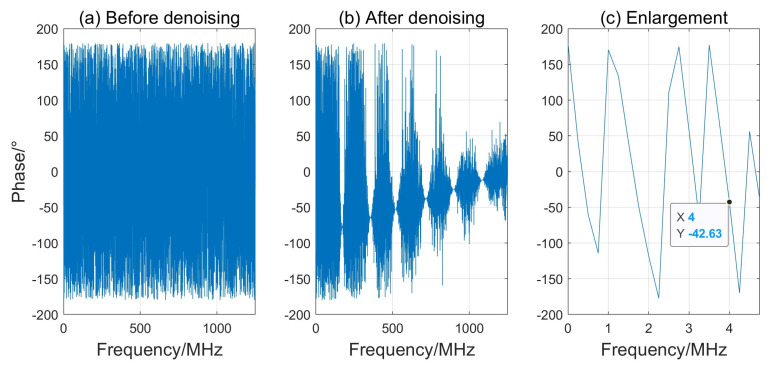
Phase spectrum (**a**) before denoising and (**b**) after denoising of echo signal from the wood specimen’s surface. (**c**) Expanded view of (**b**) within the 1–5 MHz frequency range.

The phase spectrum can be either wrapped or unwrapped. The wrapped phase spectrum limits the phase values to the range from −π to π, which helps with visualizing and analyzing phase changes, especially when there is a phase shift (such as a half-wave loss). The unwrapped phase spectrum, on the other hand, shows continuous phase changes from −∞ to ∞, which may be more intuitive in some cases but may not be as clear as the wrapped phase spectrum when analyzing phase jumps. Therefore, the wrapped phase spectrum was employed to analyze the phase of the echo signals reflected from the slag specimen surfaces. Figure 8a,b depicts the wrapped full-phase spectrum of the echo signal from the wood specimen interface before and after the denoising process. Figure 8c presents an expanded view of the denoised phase spectrum from 1 to 5 MHz, highlighting the specific frequency range of interest.

The central frequency of the denoised signal was 4 MHz, which was obtained from the IMF1 component shown in Figure 6. Then, the corresponding phase of −42.63° could be picked out from the enlargement of the denoised signal’s phase spectrum in Figure 8c. This experimental result shows that the detector’s operational frequency deviated from its nominal value of 5 MHz, which is potentially attributable to variations in crystal attachment, manufacturing tolerances, and probe aging.

The decomposition and phase analysis of PSO-VMD were performed for six different material samples, and the results are presented in Table 3. The table shows that the ultrasonic signal phase of the non-metallic materials was negative, while that of the metallic materials was positive, indicating opposite signs. Therefore, it can be concluded that the phase of the ultrasonic echo signal can reflect whether a slag inclusion is metallic or non-metallic.

In austenitic stainless steel, if the ultrasonic testing signals of slag inclusion defects in a weld show indications of half-wave loss, then this is typically indicative of tungsten inclusions. This type is a denser medium relative to the austenitic stainless steel matrix. The tungsten inclusion defect in austenitic steel mainly occurs during the process of tungsten inert gas (TIG) welding. If there is no half-wave loss in the phase change of an echo reflected from the slag interface, then this indicates that the slag inclusion is a thin medium, which is always non-metallic compounds or other metallic inclusions like aluminum particles. Identifying the category of a slag inclusion defect is critical for finding the reasons for defects developing, improving production efficiency, and reducing the rate of defective products, ultimately reducing production costs.

## 5. Conclusions

This paper designed a water environment-based simulation method for weld inclusion material classification using ultrasonic testing and introduced the PSO-VMD denoising and phase processing method with an ultrasonic signal. The classification of slag inclusion material is effectively carried out using the phase spectrum of an echo signal. To summarize this paper, we can draw the following conclusions:

- A water environment experiment with six categories of cubic samples, including four metallic and two non-metallic materials, was used to simulate the slag inclusion defects with different materials in the weldments of austenitic stainless steel.
- The proposed method with PSO-VMD denoising the ultrasonic signals utilizes PSO to optimize the VMD hyperparameters, including the suitable number of decomposition modes *K* and penalty factor α. This was proven to effectively denoise the noisy signal and to be superior to other comparative algorithms, namely WOA-VMD, GA-VMD and ALO-VMD. PSO-VMD’s results show that it decomposed the minimum number of modes (only seven IMFs) but with the highest SNR and lowest RSME among all methods, which demonstrates that PSO-VMD has superior performance in the noise reduction of slag inclusion defect detection signals.
- The phase spectrum was proposed as a valuable tool for analysis of the phase characteristics of ultrasound echo signals. By analyzing the phase spectrum, we can ascertain whether a slag inclusion has a thicker or thinner medium compared with the austenitic stainless steel base material, based on the presence or absence of a half-wave loss in the echo signal phase.

Our work provides a referential identification method for identifying material types with slag inclusion defects for practical applications.

## Figures and Tables

**Table 1:** Comparative results for parameters, evaluation index, and processing time for different VMD optimization algorithms.

Methods	WOA-VMD	GA-VMD	ALO-VMD	PSO-VMD
K	10	8	10	7
α	5000	4589	27,595	2962
SNR/dB	22.854	22.8964	22.908	23.0279
RMSE	0.02139	0.02129	0.02126	0.02097
Processing time/s	79.7	1155.9	78.9	536.7

**Table 2:** NCC between the IMF component of the denoised signal and the original signal.

IMFs	IMF1	IMF2	IMF3	IMF4	IMF5	IMF6	IMF7
NCC	0.9975	0.0330	0.0291	0.0288	0.0280	0.0275	0.0271

**Table 3:** Frequencies and phases of 6 samples’ echo signals.

Material	Frequency (MHz)	Phase (°)
Wood	4	−43.36
Resin	4	−24.81
Iron	4.25	97.11
Copper	4.25	64.87
Magnet	4.25	161.7
Aluminum Alloy	4.25	155.6

## Data Availability

Data are available upon request from the authors.

## References

[1] Kaçar R., Baylan O. (2004). An investigation of microstructure/property relationships in dissimilar welds between martensitic and austenitic stainless steels. Mater. Des..

[2] Haghshenas M., Gerlich A.P. (2018). Joining of automotive sheet materials by friction-based welding methods: A review. Eng. Sci. Technol. Int. J..

[3] Malarvel M., Singh H. (2021). An autonomous technique for weld defects detection and classification using multi-class support vector machine in X-radiography image. Optik.

[4] Dmitriev S.F., Malikov V.N., Sagalakov A.M., Shevtsova L.I. (2017). Flaw inspection of welded joints in titanium alloys by the eddy current method. Weld. Int..

[5] Abolfazl Zolfaghari A.Z., Kolahan F. (2018). Reliability and sensitivity of magnetic particle nondestructive testing in detecting the surface cracks of welded components. Nondestruct. Test. Eval..

[6] Azeez S.T., Mashinini P.M. (2022). Radiography examination of friction stir welds of dissimilar aluminum alloys. Mater. Today Proc..

[7] Dorafshan S., Maguire M., Collins W. (2018). Infrared Thermography for Weld Inspection: Feasibility and Application. Infrastructures.

[8] Hwang Y.I., Park J., Kim H.J., Song S.J., Cho Y.S., Kang S.S. (2019). Performance Comparison of Ultrasonic Focusing Techniques for Phased Array Ultrasonic Inspection of Dissimilar Metal Welds. Int. J. Precis. Eng. Manuf..

[9] Ghubade A.B., Kumar A. (2019). Review on casting defects and methodologies for quality improvement. J. Emerg. Technol. Innovat. Res..

[10] Singh R.R.B., Sasikumar T., Suresh S., Ramanan G. (2020). A Novel Detection of Defects in Al–SiC Composite by Active Pulsed Infrared Thermography Using Data and Image Processing. Trans. Indian Inst. Met..

[11] Li P., Xie S., Wang K., Zhao Y., Zhang L., Chen Z., Uchimoto T., Takagi T. (2019). A novel frequency-band-selecting pulsed eddy current testing method for the detection of a certain depth range of defects. NDT Int..

[12] Oswald-Tranta B., Schmidt R. Crack depth determination with inductive thermography. Proceedings of the Thermosense: Thermal Infrared Applications XXXVII.

[13] Wang K.Y., Cheng Y.Y. (2009). Design of X-ray digital imaging and data acquisition system. Russ. J. Nondestruct. Test..

[14] Tkocz J., Greenshields D., Dixon S. (2019). High power phased EMAT arrays for nondestructive testing of as-cast steel. NDT Int..

[15] Li X., Li B., Liu Z., Niu R., Liu Q., Huang X., Xu G., Ruan X. (2019). Detection and Numerical Simulation of Non-Metallic Inclusions in Continuous Casting Slab. Steel Res. Int..

[16] Mukhtar M.F.H., Mahmod M.F. (2021). Simulation Analysis of Ultrasonic Testing in Steel-based Butt Weld Joint. Res. Prog. Mech. Manuf. Eng..

[17] Song Y., Hua L., Wang X., Wang B., Liu Y. (2015). Research on the Detection Model and Method for Evaluating Spot Welding Quality Based on Ultrasonic A-Scan Analysis. J. Nondestruct. Eval..

[18] Kasban H., Zahran O., Arafa H., El-Kordy M., Elaraby S.M., Abd El-Samie F. (2011). Welding defect detection from radiography images with a cepstral approach. NDT Int..

[19] Ditchburn R., Burke S., Scala C. (1996). NDT of welds: State of the art. NDT Int..

[20] Daubechies I. (1990). The wavelet transform, time-frequency localization and signal analysis. IEEE Trans. Inf. Theory.

[21] Huang N.E., Shen Z., Long S.R., Wu M.C., Shih H.H., Zheng Q., Yen N.C., Tung C.C., Liu H.H. (1998). The empirical mode decomposition and the Hilbert spectrum for nonlinear and non-stationary time series analysis. Proc. R. Soc. Lond. Ser. Math. Phys. Eng. Sci..

[22] Wu Z., Huang N.E. (2009). Ensemble empirical mode decomposition: A noise-assisted data analysis method. Adv. Adapt. Data Anal..

[23] Torres M.E., Colominas M.A., Schlotthauer G., Flandrin P. A complete ensemble empirical mode decomposition with adaptive noise. Proceedings of the 2011 IEEE International Conference on Acoustics, Speech and Signal Processing (ICASSP).

[24] Dragomiretskiy K., Zosso D. (2014). Variational Mode Decomposition. IEEE Trans. Signal Process..

[25] Li H., Liu T., Wu X., Chen Q. (2020). An optimized VMD method and its applications in bearing fault diagnosis. Measurement.

[26] Wang D., Yue C., Wei S., Lv J. (2017). Performance Analysis of Four Decomposition-Ensemble Models for One-Day-Ahead Agricultural Commodity Futures Price Forecasting. Algorithms.

[27] Hua T., Dai K., Zhang X., Yao Z., Wang H., Xie K., Feng T., Zhang H. (2019). Optimal VMD-Based Signal Denoising for Laser Radar via Hausdorff Distance and Wavelet Transform. IEEE Access.

[28] Qi B., Yang G., Guo D., Wang C. (2021). EMD and VMD-GWO parallel optimization algorithm to overcome Lidar ranging limitations. Opt. Express.

[29] Long J., Wang X., Dai D., Tian M., Zhu G., Zhang J. (2017). Denoising of UHF PD signals based on optimised VMD and wavelet transform. IET Sci. Meas. Technol..

[30] Eberhart R., Kennedy J. A new optimizer using particle swarm theory. Proceedings of the MHS’95 Sixth International Symposium on Micro Machine and Human Science.

[31] Richman J.S., Lake D.E., Moorman J. (2004). Sample Entropy. Numerical Computer Methods, Part E.

[32] Cao M., Yuan J., Liu H., Fang X., Zhu J. (2003). A simulation of the quasi-standing wave and generalized half-wave loss of electromagnetic wave in non-ideal media. Mater. Des..

[33] Wojciech J., Jacek G. (2021). Detection of slag inclusions using infrared thermal imagining system. MATEC Web Conf..

[34] Liu Z., Peng Y. (2023). Study on Denoising Method of Vibration Signal Induced by Tunnel Portal Blasting Based on WOA-VMD Algorithm. Appl. Sci..

[35] Liu Z., Liu H. (2023). A novel hybrid model based on GA-VMD, sample entropy reconstruction and BiLSTM for wind speed prediction. Measurement.

[36] Shi L., Wen J., Pan B., Xiang Y., Zhang Q., Lin C. (2020). Dynamic Characteristics of a Gear System with Double-Teeth Spalling Fault and Its Fault Feature Analysis. Appl. Sci..

